# Checkpoint based immunotherapy in non-small cell lung cancer: a real-world retrospective study

**DOI:** 10.3389/fimmu.2024.1419544

**Published:** 2024-11-27

**Authors:** Luigi Liguori, Gabriele Giorgio, Giovanna Polcaro, Valentina Pagliara, Domenico Malandrino, Francesco Perri, Marco Cascella, Alessandro Ottaiano, Valeria Conti, Alberto Servetto, Roberto Bianco, Stefano Pepe, Francesco Sabbatino

**Affiliations:** ^1^ Department of Medicine, Surgery and Dentistry “Scuola Medica Salernitana”, University of Salerno, Baronissi, Italy; ^2^ Department of Clinical Medicine and Surgery, University of Naples Federico II, Naples, Italy; ^3^ Medical and Experimental Head and Neck Oncology Unit, Istituto Nazionale Tumori IRCCS Fondazione G. Pascale, Naples, Italy; ^4^ Division of Innovative Therapies for Abdominal Metastases, Istituto Nazionale Tumori IRCCS Fondazione G. Pascale, Naples, Italy

**Keywords:** biomarker, ICI, immunotherapy, NSCLC, PD-1, PD-L1, predictive, real-world

## Abstract

**Introduction:**

Immune checkpoint inhibitor (ICI)-based immunotherapy targeting programmed cell death 1 (PD-1) or its ligand 1 (PD-L1) has radically changed the management of many types of solid tumors including non-small cell lung cancer (NSCLC). Many clinical trials have demonstrated that ICIs improve the survival and the quality of life of patients with advanced non oncogene NSCLC as compared to standard therapies. However, not all patients achieve a clinical benefit from this immunotherapeutic approach. As a result, real-word validation of the efficacy and safety of ICIs can be useful for defining potential predictive biomarkers as well as for overcoming limitations linked to clinical trial restrictions.

**Methods:**

We retrospectively retrieved the clinical data of patients with advanced non oncogene NSCLC treated with ICIs (anti-PD-1 or anti-PD-L1) as single agent or in combination with chemotherapy at “San Giovanni di Dio e Ruggi D’Aragona” University Hospital from January 2016 to December 2023. Potential correlations between clinical-pathological characteristics and safety or survival outcomes were investigated employing the Fisher’s exact test, Mann-Whitney U test, the Kruskal-Wallis method and log-rank test, as applicable. Multivariate survival analyses were performed using the Cox proportional hazards model.

**Results:**

Clinical data of 129 patients were retrieved. At a median follow-up of 29.70 months, progression-free survival (PFS) and overall survival (OS) were 5.27 months and 8.43 months, respectively. At the multivariate analyses, smoking status, presence of bone metastases and the occurrence of immune-related adverse events (irAEs) were correlated with both PFS and OS. Moreover, patients treated with anti-PD-1-based therapy achieved an increased clinical benefit than those treated with anti-PD-L1.

**Discussion:**

In this study we described our real-world experience of ICIs for the treatment of patients with advanced non oncogene NSCLC. A decreased OS in our study population was reported as compared to that of patients included in the clinical trials. Noteworthy, correlations between clinical-pathological characteristics and survival outcomes emerged. Nevertheless, the potential integration of clinical-pathological characteristics as predictive biomarkers in more accurate therapeutic algorithms as well as the underlying biological mechanisms should be further validated in ad hoc studies.

## Introduction

Lung cancer, mainly represented by non-small cell lung cancer (NSCLC), is the leading cause of cancer-related death in USA, with about 120.000 deaths per year (1). In the past few years, immune checkpoint inhibitors (ICIs) targeting programmed cell death 1 (PD-1), its ligand 1 (PD-L1) and cytotoxic T-lymphocyte antigen-4 (CTLA-4) have revolutionized the management of many types of solid tumors including NSCLC (2). This novel immunotherapeutic approach has demonstrated to improve the survival and the quality of life of the patients with advanced non oncogene NSCLC as compared to standard chemotherapy (2). Based on the results of many clinical trials, ICIs as monotherapy or in combination with chemotherapy represent the standard-of-care for the treatment of patients with advanced non oncogene NSCLC, so far (2). However, not all treated patients achieved a sustained clinical benefit. Indeed, the efficacy of this therapy is limited to an half of treated patients, and only a small portion of them (10-15%) achieves long-term tumor response (3–9, 11). Moreover, about 10-15% of treated patients develops severe immune-related adverse events (irAEs), potentially causing prolonged sequelae or even fatal consequences (3–9, 11). As a result, there is the urgent need to identify biomarkers of tumor response as well as patients at higher risk to develop severe irAEs. In the last decade, several pathological biomarkers including PD-L1 tumor proportion score (TPS), tumor mutational burden (TMB), human leucocyte antigen (HLA) class I and II expression, β2-microglobulin (β2m) mutations, tumor microenvironment (TME) composition, and gene expression profiles (GEPs) have been investigated with various results (12–16). PD-L1 TPS, the most widely investigated, is integrated in therapeutic algorithm currently utilized in clinical practice for the treatment of advanced non oncogene NSCLC patients. Indeed, increased levels of PD-L1 TPS are correlated to a higher likelihood of tumor response (6, 11, 13, 17). However, not all patients with high PD-L1 TPS achieve a clinical benefit. In addition, even patients with low or negative PD-L1 TPS may also benefit from this therapy. Consequently, PD-L1 TPS is not efficient in predicting tumor response, being considered a “surrogate biomarker” (13, 17–19). On the same line, no other biomarker has demonstrated to efficiently predict either tumor response and/or development of irAEs (12–15).

Beyond pathological biomarkers, many clinical characteristics including gender, Eastern Cooperative Oncology Group (ECOG) Performance Status (PS), Body Mass Index (BMI), specific sites of metastases, concomitant medications (i.e. antibiotics and corticosteroids) and occurrence of irAEs have also been investigated for their potential predictive role with various results (20). Here by analyzing real-world data we aim to further validate the efficacy and safety of ICIs in study populations by defining potential predictive biomarkers as well as by overcoming limitations linked to clinical trial restrictions.

## Materials and methods

### Study population

Clinical data of Caucasian patients with confirmed advanced (stage IV) NSCLC treated with ICIs from January 2016 to December 2023 at “San Giovanni di Dio e Ruggi D’Aragona” University Hospital, was retrieved. The study was performed without interfering with clinical practice. Selection of patients to be included in the study was performed based on: (i) signed informed consent for clinical-pathological data acquisition; (ii) age >18 years; (iii) treatment with ICIs as monotherapy or in combination with chemotherapy; (iv) absence of active autoimmune disease. Patients with Epidermal Growth Factor Receptor (EGFR), Anaplastic Lymphoma Kinase (ALK), c-ros oncogene 1 (ROS1), V-Raf Murine Sarcoma Viral Oncogene Homolog B (BRAF), Mesenchymal-epithelial transition factor (MET), REarranged during Transfection (RET) and Neurotrophic Tropomyosin Receptor Kinases (NTRK) tumor alterations were excluded from the study. Evaluation of ALK, BRAF, EGFR, MET, NTRK, RET and ROS1 tumor alterations was performed on tumor samples (when available) or liquid biopsy according to national pathology guidelines. Clinical-pathological characteristics including age, sex, ECOG PS, smoking status, alcohol abuse, comorbidities, previous cancer, concomitant medications, baseline prednisone equivalent dose, PD-L1 TPS, histologic subtypes, specific sites of metastasis, type of immunotherapy and previous chemotherapy and/or targeted therapy and/or radiotherapy were retrospectively collected. Patient privacy and personal data were preserved by assigning a progressive anonymous identification number. PD-L1 was evaluated on tumor samples as clinically indicated when tumor tissue was available and reported as TPS (21) according to European Society for Medical Oncology (ESMO) guidelines. Patients received one of the following ICI-based immunotherapy according to Italian guidelines: i) atezolizumab or nivolumab after the failure of platinum-based chemotherapy, regardless PD-L1 TPS; ii) pembrolizumab after the failure of platinum-based chemotherapy for PD-L1 TPS ≥1%; iii) the combination of chemotherapy and pembrolizumab or the combination of chemotherapy and nivolumab and ipilimumab as first-line for PD-L1 TPS <50%; iv) atezolizumab or pembrolizumab as first-line for PD-L1 TPS ≥50%;. irAEs were defined as adverse events displaying a certain, likely or possible correlation with ICIs according to Common Terminology Criteria for Adverse Events (CTCAE) v 4.0 (22). Radiographic imaging was performed every two months, according to clinical practice. Response rate was determined according to Response Evaluation Criteria in Solid Tumours version 1.1 (RECIST v1.1) (23) and reported as complete response (CR), partial response (PR), stable disease (SD) and progression disease (PD). Objective response rate (ORR) was defined as the proportion of patients with a CR or PR whereas disease control rate (DCR) as the proportion of patients with CR or PR or SD. Progression-free survival (PFS) was defined as the time from the start of the treatment to the first documented PD or death by any cause. Overall survival (OS) was defined from the start of the treatment to death by any cause or last follow-up date. Patients dead from COVID-19 were excluded. The study was approved by the local ethics committee (prot./SCCE n.85275), in accordance with the Declaration of Helsinki and its amendments.

### Statistical analysis

Data was collected using Microsoft Excel. Statistical analyses were performed using STATA v13 software released by StataCorp LP (College Station, TX, USA). Continuous variables were expressed as medians and ranges, whereas categorical variables were expressed as frequencies and percentages. PFS and OS were calculated using the Kaplan-Meier method. Median follow-up was calculated using the inverse Kaplan-Meier method. Correlations between clinical-pathological characteristics and irAE rates were performed using the Fisher’s exact test, Mann–Whitney U test and the Kruskal-Wallis method, as appropriate. Correlation between clinical-pathological characteristics and survival outcomes (PFS and OS) was performed using log-rank test. Multivariate survival analyses were performed using the Cox proportional hazards model. The difference between groups was considered significant when the P value was <0.05.

## Results

### Clinical-pathological characteristics of NSCLC patients treated with ICIs

Clinical-pathological characteristics of 129 Caucasian patients with stage IV non oncogene NSCLC at the “San Giovanni di Dio e Ruggi D’Aragona” University Hospital, treated with ICIs from January 2016 to December 2023 were retrieved. Baseline characteristics of patients are summarized in **Table 1**.

**Table 1:** Baseline clinical-pathological characteristics of patients included in the study.

Median age 68 years (range, 45-83 years)	
Sex	
Male Female	100 (77.52%) 29 (22.48%)
ECOG PS	
0 1 2 3	47 (36.43%) 58 (44.96%) 19 (14.73%) 5 (3.88%)
Alcohol abuse	
No Yes	119 (92.25%) 10 (7.75%)
Smoking status	
Never smoker Previous smoker Current smoker	14 (10.85%) 77 (59.69%) 38 (29.46%)
Comorbidities	
Hypertension Dyslipidemia Diabetes COPD HF Depressive disorder CRF	69 (53.49%) 36 (27.90%) 26 (20.16%) 22 (17.05%) 10 (7.75%) 4 (3.10%) 1 (0.77%)
Previous cancer Prostate cancer Breast cancer	14 (10.85%) 13 (92.86%) 1 (7.14%)
Concomitant medications	
Anticoagulants Antiplatelet drugs Antihypertensive Oral hypoglycemic drugs Statins Antidepressants Opioids Low dose aspirin	24 (18.60%) 24 (18.60%) 69 (53.49%) 19 (14.73%) 32 (24.81%) 11 (8.53%) 33 (25.58%) 41 (31.78%)
Baseline prednisone equivalent dose	
No treatment ≤10 mg/die >10 mg/die ≤20 mg/die >20 mg/die	65 (50.39%) 16 (12.40%) 28 (21.71%) 20 (15.50%)
PD-L1 TPS	
NA <1% ≥1% <50% ≥50%	31 (24.03%) 37 (28.68%) 31 (24.03%) 30 (23.26%)
Histology	
Adenocarcinoma Squamous cell carcinoma Large cell carcinoma Sarcomatoid carcinoma	80 (62.02%) 44 (34.11%) 4 (3.10%) 1 (0.77%)
Sites of metastasis	
Lymph node Lung Bone CNS Adrenal gland Liver Skin	112 (86.82%) 84 (65.12%) 38 (29.46%) 28 (21.71%) 23 (17.83%) 15 (11.63%) 6 (4.65%)
ICI-line of treatment	
First-line Second-line Third-line Fourth-line Fifth-line Sixth-line	44 (34.11%) 36 (27.91%) 38 (29.46%) 8 (6.20%) 2 (1.55%) 1 (0.77%)
Previous chemotherapy	
Yes No	60 (46.51%) 69 (53.49%)
Previous anti-VEGFR targeted therapy	
Yes No	4 (3.10%) 125 (96.90%)
Previous radiotherapy	
Yes No	39 (30.23%) 90 (69.77%)
Type of immunotherapy	
Nivolumab Pembrolizumab Atezolizumab Carboplatin-pemetrexed-pembrolizumab Carboplatin-nab-paclitaxel- pembrolizumab Carboplatin-pemetrexed- ipilimumab-nivolumab	45 (34.87%) 25 (19.37%) 9 (6.98%) 38 (29.46%) 7 (5.43%) 5 (3.88%)
Median number of ICI cycle received	15 (1–123)
Type of response	
CR PR SD PD ORR DCR	1 (0.96%) 20 (19.23%) 42 (40.38%) 41 (39.43%) 21 (20.19%) 63 (60.57%)
Pseudoprogression	
Yes No	2 (1.96%) 100 (98.04%)
Survival outcomes	
Median follow-up Median PFS Median OS	15.00 months (range, 1.20-93.23) 5.27 months (range, 0.30-93.23) 8.43 months (range, 0.30-93.23)

CNS, central nervous system; COPD, Chronic obstructive pulmonary disease; CR, complete response; CRF, chronic renal failure; DCR, disease control rate; ECOG, Eastern Cooperative Oncology Group; HF, heart failure; ICI, immune checkpoint inhibitor; NA, not available; ORR, objective response rate; OS, overall survival; PD, progression disease; PD-L1, programmed death-ligand 1; PFS, progression-free survival; PR, partial response; PS, Performance Status; SD, stable disease; TPS, tumor proportion score; VEGFR, Vascular Endothelial Growth Factor Receptor.

**Figure 1:**
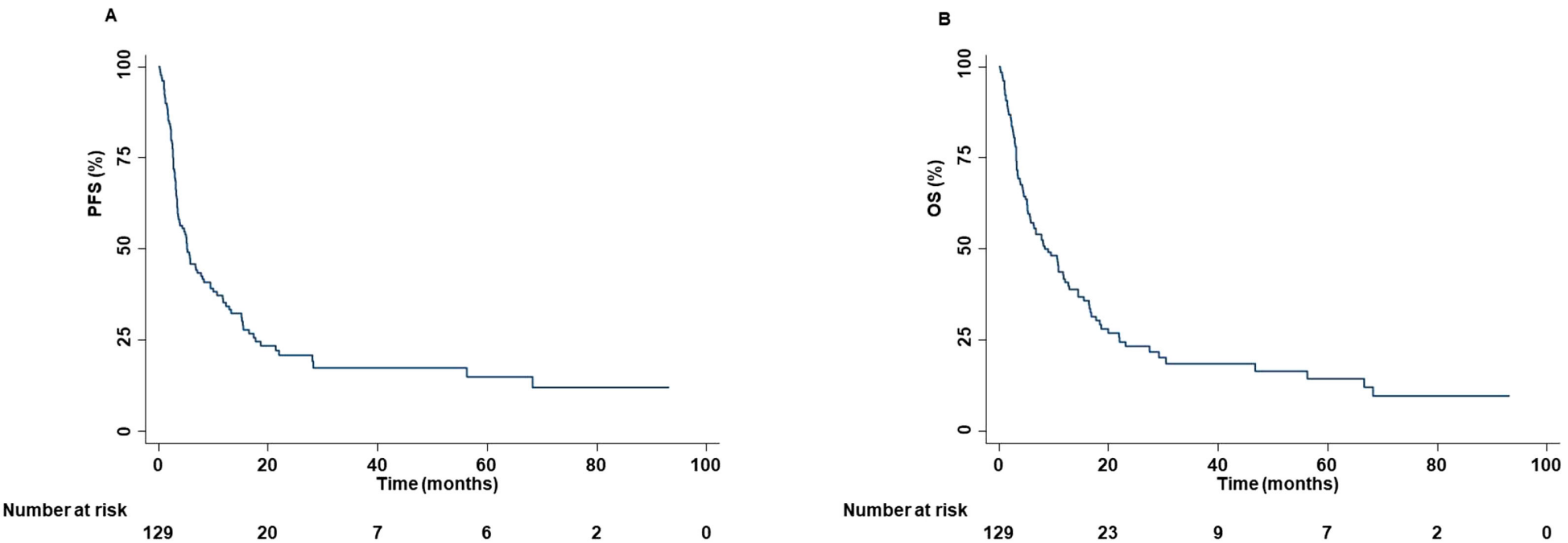
PFS and OS of advanced non oncogene NSCLC patients treated with ICI-based immunotherapy. At a median follow-up of 15.00 months, median PFS and OS were 5.27 months (range, 0.30-93.23 months) **(A)** and 8.43 months (range, 0.30-93.23 months) **(B)**, respectively. PFS and OS analysis was performed using the Kaplan-Meier method.

The median age was 68 years (range, 45-83 years). One hundred patients (77.52%) were male. Forty-seven (36.43%), 58 (44.96%), 19 (14.73%), and 5 (3.88%) had ECOG PS of 0, 1, 2 and 3, respectively. Fourteen patients (10.85%) were never smokers, while 77 (59.69%) and 38 (29.46%) were previous and current smokers, respectively. Alcohol abuse was also reported in ten patients (7.75%). Relevant comorbidities included hypertension (53.49%), dyslipidemia (27.90%), diabetes (20.16%), chronic obstructive pulmonary disease (COPD) (17.05%), hearth failure (7.75%), depressive disorder (3.10%) and chronic renal failure (CRF) (0.77%). Thirteen and one patients reported a previous prostate cancer and breast cancer, respectively. Relevant concomitant medications included antihypertensive (53.49%), low dose aspirin (31.78%), opioids (25.78%), statins (24.81%), anticoagulants (18.60%), antiplatelet drugs (18.60%), oral hypoglycemic drugs (14.73%), and antidepressants (selective serotonin reuptake inhibitors (SSRIs)) (8.53%). In addition, 16, 28 and 20 patients received equivalent prednisone dose of ≤10 mg/die, >10 mg/die and ≤20 mg/die, and >20 mg/die, respectively. Eighty tumors (62.02%) were classified as adenocarcinomas, 44 (34.11%) as squamous cell carcinoma, 4 (3.10%) as large cell carcinoma and 1 (0.77%) as sarcomatoid carcinoma. PD-L1 TPS was available for 98 patients (75.97%). A PD-L1 TPS <1%, ≥1% and <50%, and ≥50% were reported in 37.76%, 31.63% and 30.61%, respectively, of the available tumors. Main sites of metastasis included lymph nodes (87.50%), lung (65.63%), bone (29.69%), central nervous system (CNS) (21.88%), adrenal gland (17.97%), liver (11.72%) and skin (4.69%). Sixty patients (46.51%), 4 (3.10%), and 39 (30.23%) of patients had previously received chemotherapy, anti-Vascular Endothelial Growth Factor Receptor (VEGFR)-targeted therapy and radiotherapy, respectively. Forty-four (34.11%), 36 (27.91%), 38 (29.46%), and 11 (8.53%) patients received ICI as first, second, third, and fourth or subsequent lines of treatment, respectively. More in detail, i) forty-five (34.87%) patients received nivolumab; ii) twenty-five (19.37%) patients received pembrolizumab; iii) nine (6.98%) patients received atezolizumab; iv) thirty-eight (28.68%) patients received carboplatin-pemetrexed-pembrolizumab; v) seven (5.43%) patients received carboplatin-nab-paclitaxel-pembrolizumab, and vi) five (3.88%) patients received carboplatin-pemetrexed-ipilimumab-nivolumab. The median cycle of ICI received was 15 (1–123). ORR was 20.19% while DCR was 60.57%. CRs, PRs, SDs, and PDs were reported in 1 (0.96%), 20 (19.23%), 42 (40.38%), and 41 (39.43%) of treated patients, respectively. In addition, pseudo-progression was also reported in two patients (1.96%). At a median follow-up of 15.00 months, median PFS and OS were 5.27 months (range, 0.30-93.23 months) and 8.43 months (range, 0.30-93.23 months), respectively (**Figure 1**).

Moreover, among patients treated with ICI as first-line of treatment, at a median follow-up of 21.30 months, the median PFS and OS were 5.87 months (range, 0.30-75.73 months) and 11.80 months (range, 0.30-75.73 months), respectively (**Supplementary Figure S1**). On the other hand, among patients treated with ICI as second or subsequent lines of treatment, at a median follow-up of 69.80 months, the median PFS and OS were 3.90 months (range, 0.47-93.23 months) and 8.12 months (range, 0.80-93.23 months), respectively (**Supplementary Figure S2**). We also evaluated the survival outcomes of patients treated with the combination of chemotherapy and ICI as well as of those treated with ICI as monotherapy. Specifically, in the former, at a median follow-up of 23.40 months, median PFS and median OS were 8.37 months (range, 0.30-35.00 months) and 11.80 months (range, 0.30-35.00 months), respectively (**Supplementary Figure S3**). Whereas, in the latter, at a median follow-up of 69.80 months, median PFS and median OS were 4.43 months (range, 0.33-93.23 months) and 8.17 months (range, 0.33-93.23 months), respectively (**Supplementary Figure S4**).

Lastly, the safety profile was also described. All grade (grade 1-2 and/or grade 3-4), grade 1-2 and grade 3-4 irAEs were reported in 81 (62.79%), 80 (62.02%) and 12 (9.38%) of treated patients, respectively. All irAEs were reported in detail in **Table 2**.

**Table 2:** Immune-related adverse events reported in the study population.

	Grade 1-2	Grade 3-4
Any event	80 (62.02%)	12 (9.38%)
Led to discontinuation of treatment	0 (0.00%)	7 (58.34%)
Adrenal insufficiency	12 (15.00%)	0 (0.00%)
Amylase increase	4 (5.00%)	1 (8.33%)
Arthritis	4 (5.00%)	0 (0.00%)
Asthenia	32 (40.00%)	2 (16.67%)
Creatinine increase	5 (6.25%)	0 (0.00%)
Decreased appetite	11 (13.75%)	2 (16.67%)
Diarrhea	11 (13.75%)	1 (8.33%)
Hepatitis	4 (5.00%)	0 (0.00%)
Hypophysitis	2 (2.50%)	0 (0.00%)
Lipase increase	2 (2.50%)	1 (8.33%)
Nausea	12 (15.00%)	1 (8.33%)
Pancreatitis	2 (2.50%)	1 (8.33%)
Pneumonitis	2 (2.50%)	5 (41.67%)
Rash	7 (8.75%)	0 (0.00%)
Stipsis	10 (12.50%)	0 (0.00%)
Thyroiditis	13 (16.25%)	0 (0.00%)
Vomiting	7 (8.75%)	0 (0.00%)

The most frequently reported irAEs of grade 1-2 and grade 3-4 were asthenia (24.81%) and pneumonitis (3.88%), respectively. Seven (5.42%) patients discontinued anti-PD-1/PD-L1 therapy because of irAEs. No treatment-related death was reported.

### Associations between clinical-pathological characteristics and survival outcomes

**Figure 2:**
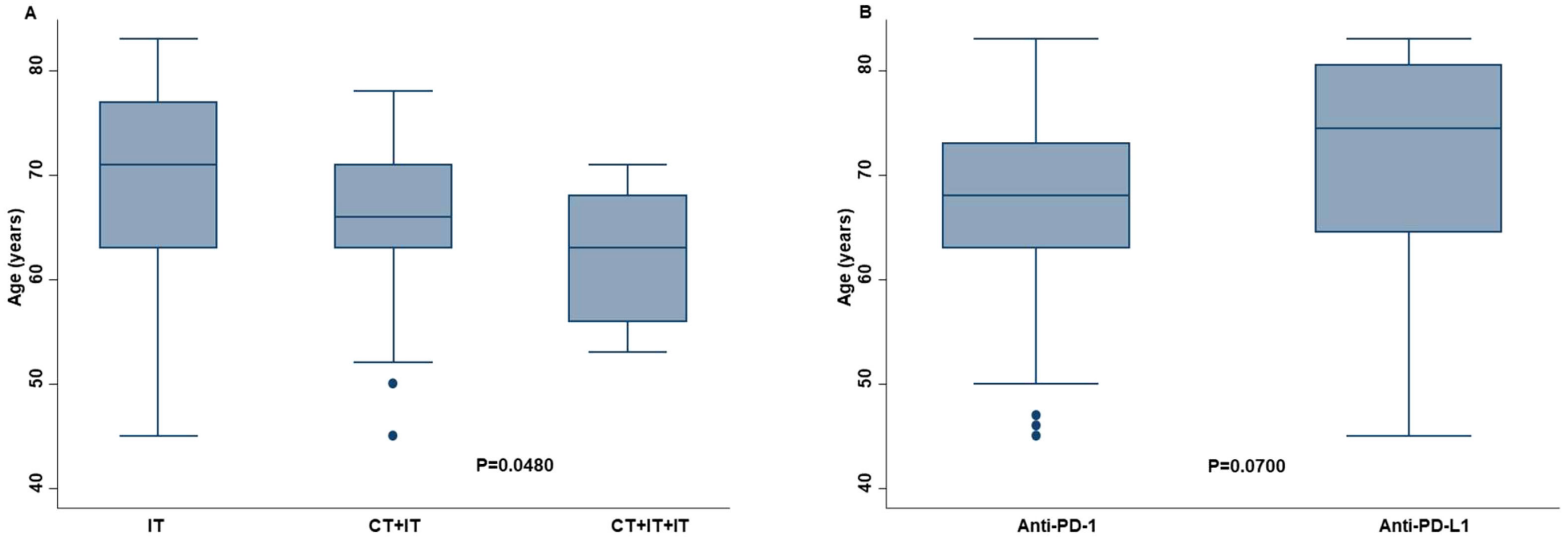
Correlation between age of the advanced non oncogene NSCLC patients and the chosen type of ICI-based immunotherapy. Older patients received more ICI as monotherapy (IT) than younger patients **(A)**. The former received less frequently the combination of chemotherapy and mono (CT+IT) or double (CT+IT+IT) ICI. Older patients also received more frequently anti-PD-L1 therapy than anti-PD-1 therapy **(B)**. Differences between groups were correlated by Kruskal-Wallis method. P <0.05 was considered statistically significant.

Age was significantly correlated with the type of therapy. Specifically, older patients received ICI as monotherapy more frequently than the combination of chemotherapy and ICI (P=0.0480) (**Figure 2A**). However, the sample size of our study population was not enough to demonstrate this association (effect size: 0.0473; power: 0.0832). In addition, older patients received anti-PD-L1 therapy more frequently than anti-PD-1 therapy (P=0.0700) (**Figure 2B**).

**Figure 3:**
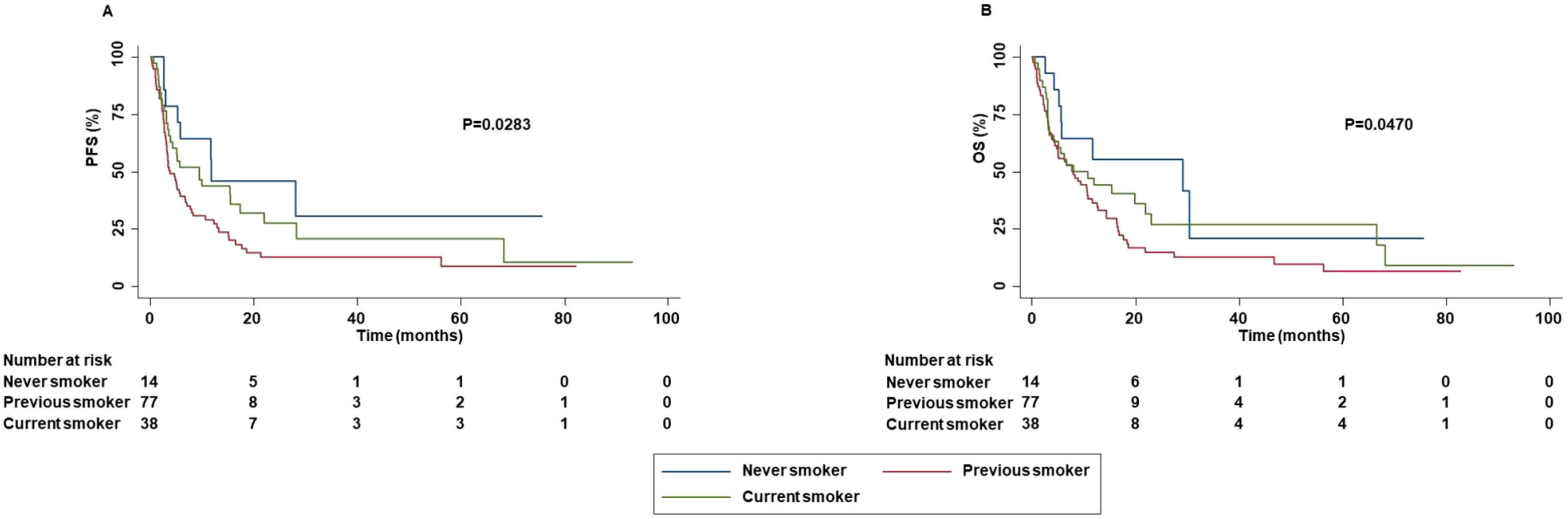
Association between smoking status and clinical outcomes in advanced non oncogene NSCLC patients treated with ICI-based immunotherapy. PFS **(A)** and OS **(B)** of advanced NSCLC patients treated with ICI-based immunotherapy were stratified based on smoking status. PFS and OS were compared using the Kaplan-Meier method. Differences in patients’ survival were analyzed using a log-rang test. P <0.05 was considered statistically significant.

Significant correlations between clinical-pathological characteristics and survival outcomes were found. PFS and OS were significantly correlated with ORR (P=0.0000 and P=0.0000) and DCR (P=0.0000 and P=0.0000). In addition, survival outcomes were also correlated with the number of ICI cycles received by the patients. Indeed, patients with longer PFS and OS received a higher number of ICI cycles (P=0.0000 and P=0.0000) than those who received a lower number. Smoking status, concomitant medications (antidepressants or opioids) and ECOG PS, were correlated with survival outcomes. Smoking status correlated with PFS (P=0.0283) and OS (P=0.0470). Specifically, patients who were never smoked or current smokers displayed an increased PFS, OS than those who were previously smokers (**Figure 3**).

**Figure 4:**
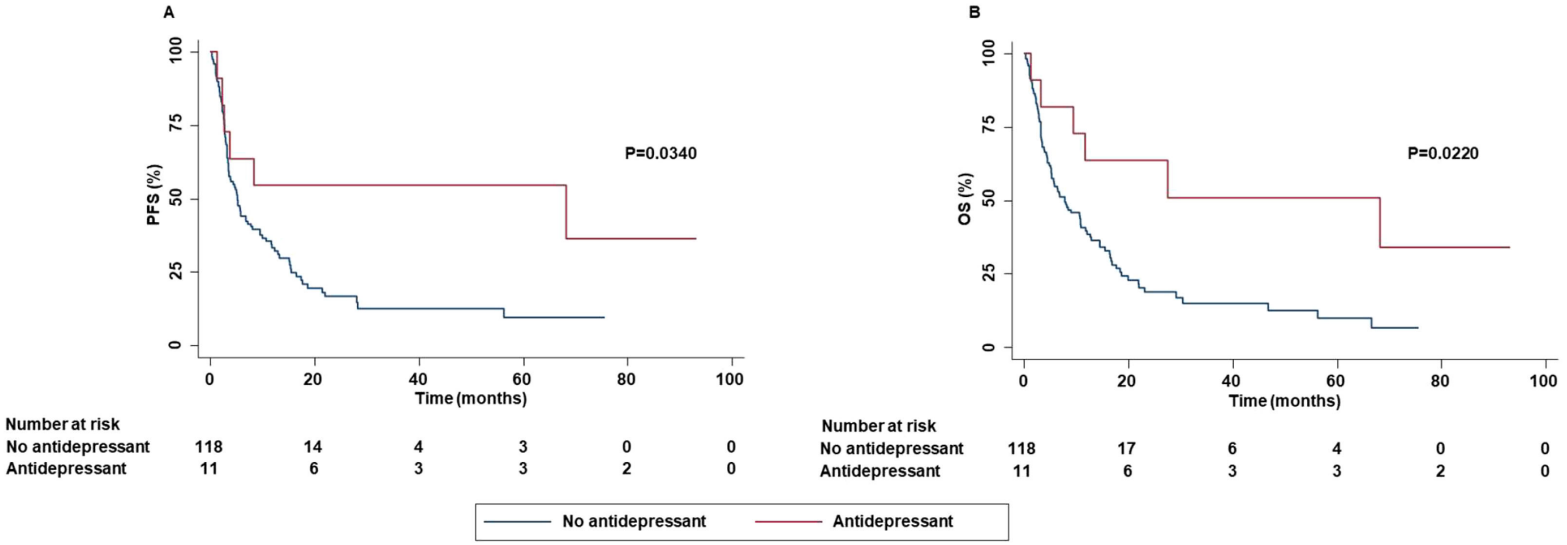
Association between concomitant administration of antidepressant and clinical outcomes in advanced NSCLC patients treated with ICI-based immunotherapy. PFS **(A)** and OS **(B)** of advanced NSCLC patients treated with ICI-based immunotherapy were stratified based on concomitant administration of antidepressant. PFS and OS were compared using the Kaplan-Meier method. Differences in patients’ survival were analyzed using a log-rang test. P <0.05 was considered statistically significant.

In addition, smoker patients achieved an increased DCR than patients who had stopped smoking. Concomitant administration of antidepressants was significantly correlated with increased PFS (P=0.0340) and OS (P=0.0220) (**Figure 4**).

**Figure 5:**
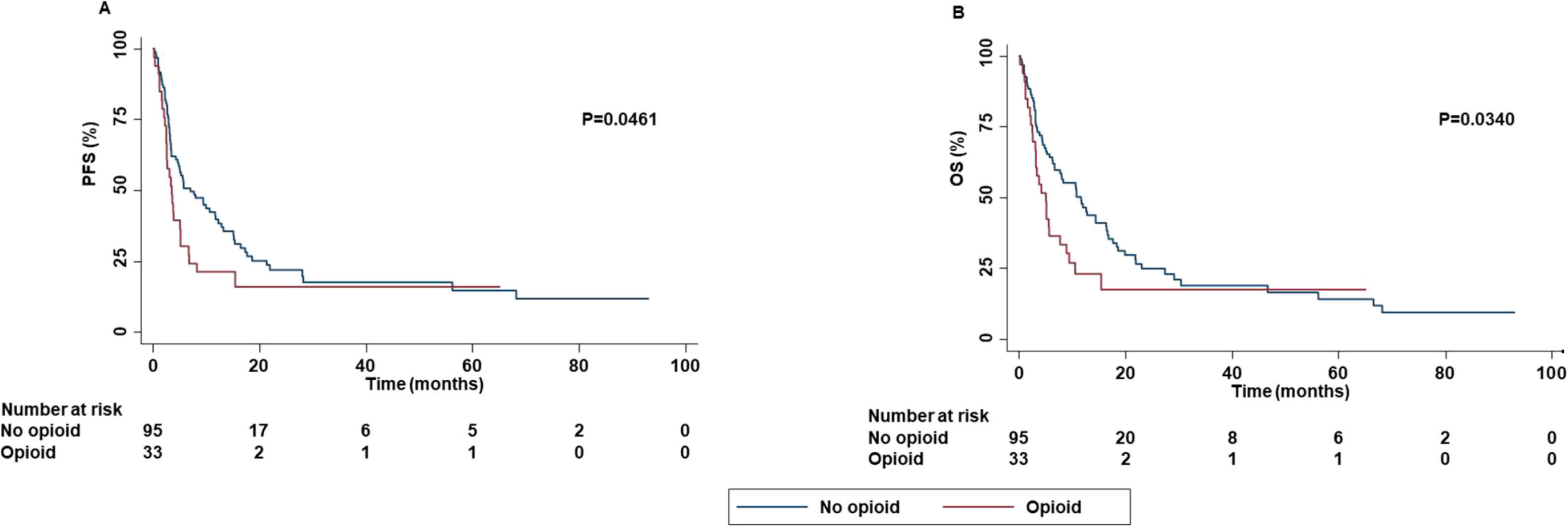
Association between concomitant administration of opioid and clinical outcomes in advanced non oncogene NSCLC patients treated with ICI-based immunotherapy. PFS **(A)** and OS **(B)** of advanced NSCLC patients treated with ICI-based immunotherapy were stratified based on concomitant administration of opioid. PFS and OS were compared using the Kaplan-Meier method. Differences in patients’ survival were analyzed using a log-rang test. P <0.05 was considered statistically significant.

In contrast, concomitant administration of opioids was significantly correlated with decreased PFS (P=0.0461), OS (P=0.0340) (**Figure 5**) while no statistically significant correlation between concomitant administration of opioids and ORR (P=0.0500) and DCR (P=0.0910) was found.

**Figure 6:**
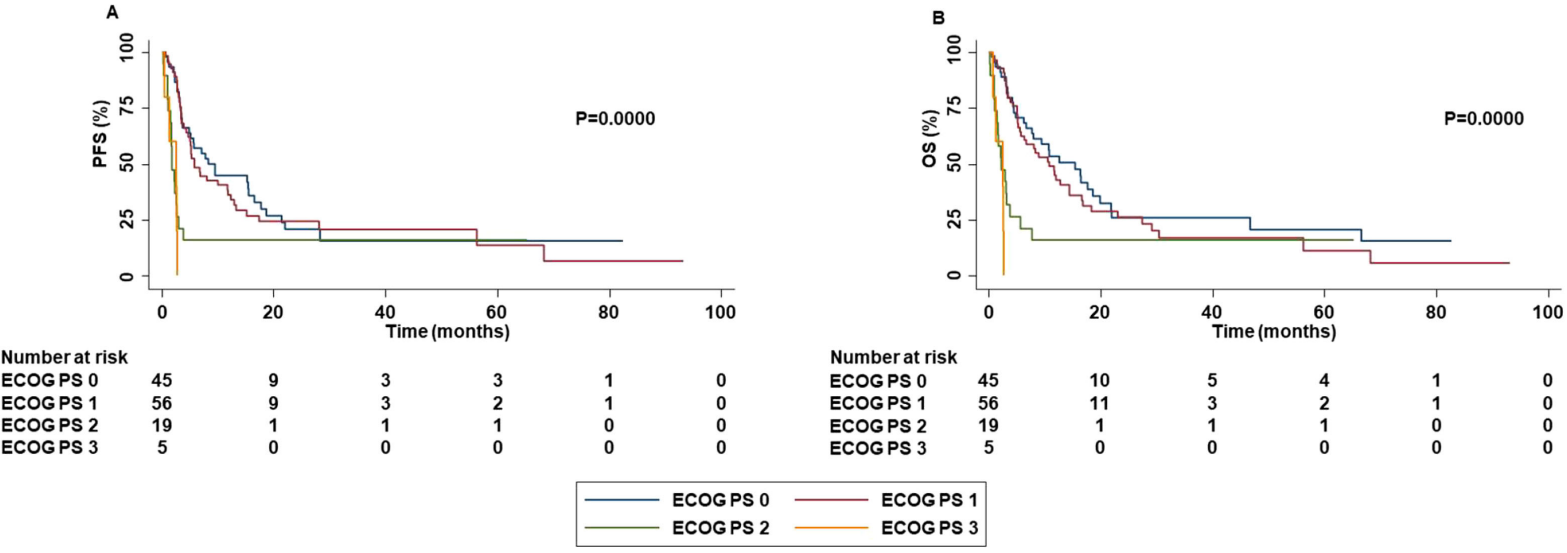
Association between ECOG PS and clinical outcomes in advanced non oncogene NSCLC patients treated with ICI-based immunotherapy. PFS **(A)** and OS **(B)** of advanced NSCLC patients treated with ICI-based immunotherapy were stratified based ECOG PS. PFS and OS were compared using the Kaplan-Meier method. Differences in patients’ survival were analyzed using a log-rang test. P <0.05 was considered statistically significant.

Patients with ECOG PS 0-1 were strongly associated with increased PFS (P=0.0000), OS (P=0.0000), ORR (P=0.0481) and DCR (P=0.0100) than those with ECOG PS 2-3 (**Figure 6**).

**Figure 7:**
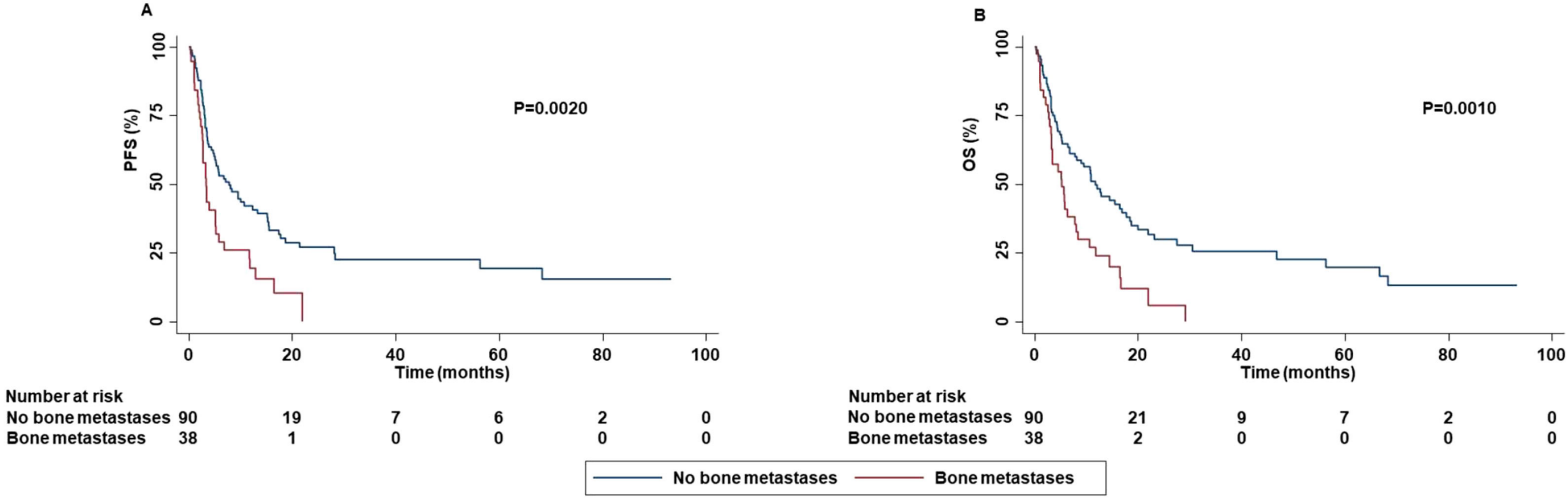
Association between bone metastases and clinical outcomes in advanced non oncogene NSCLC patients treated with ICI-based immunotherapy. PFS **(A)** and OS **(B)** of advanced NSCLC patients treated with ICI-based immunotherapy were stratified based on the presence of bone metastases. PFS and OS were compared using the Kaplan-Meier method. Differences in patients’ survival were analyzed using a log-rang test. P <0.05 was considered statistically significant.

Patients with bone metastases displayed a decreased PFS (P=0.0020), OS (P=0.0010) and DCR (P=0.0240) than those without bone metastases (**Figure 7**).

**Figure 8:**
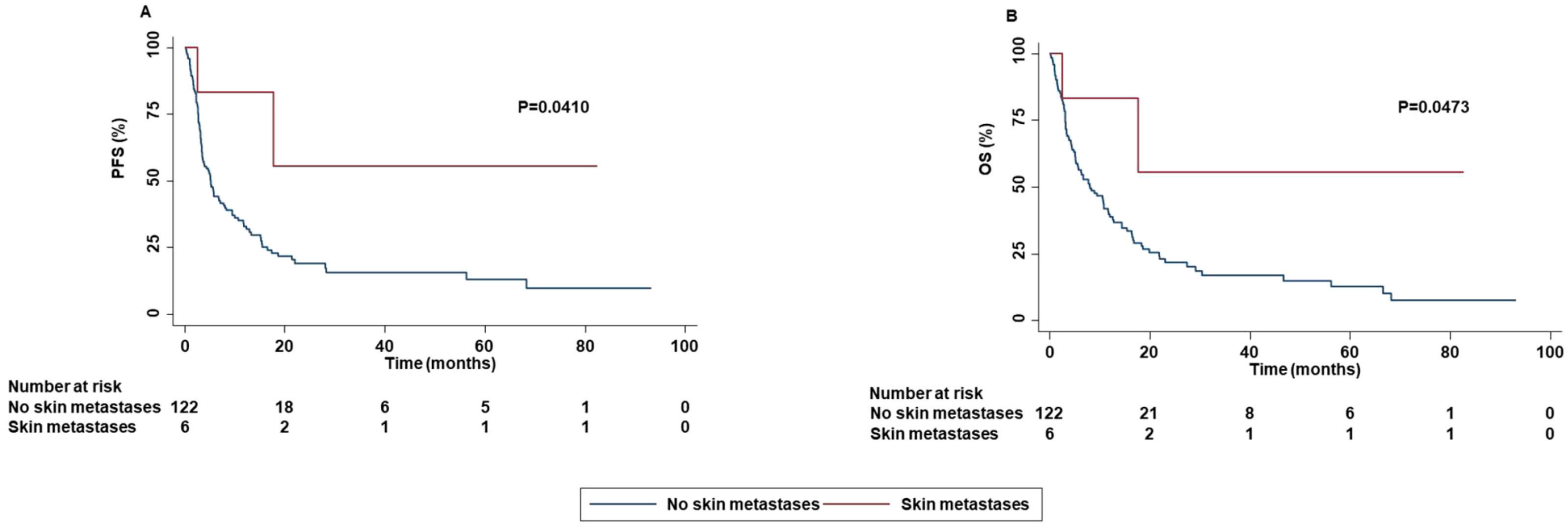
Association between skin metastases and clinical outcomes in advanced oncogene NSCLC patients treated with ICI-based immunotherapy. PFS **(A)** and OS **(B)** of advanced NSCLC patients treated with ICI-based immunotherapy were stratified based on the presence of skin metastases. PFS and OS were compared using the Kaplan-Meier method. Differences in patients’ survival were analyzed using a log-rang test. P <0.05 was considered statistically significant.

In contrast in patients with skin metastases an increased PFS (P=0.0410) and OS (P=0.0473) as well as a higher ORR (P=0.0500) were reported as compared to that of patients without skin metastases (**Figure 8**).

**Figure 9:**
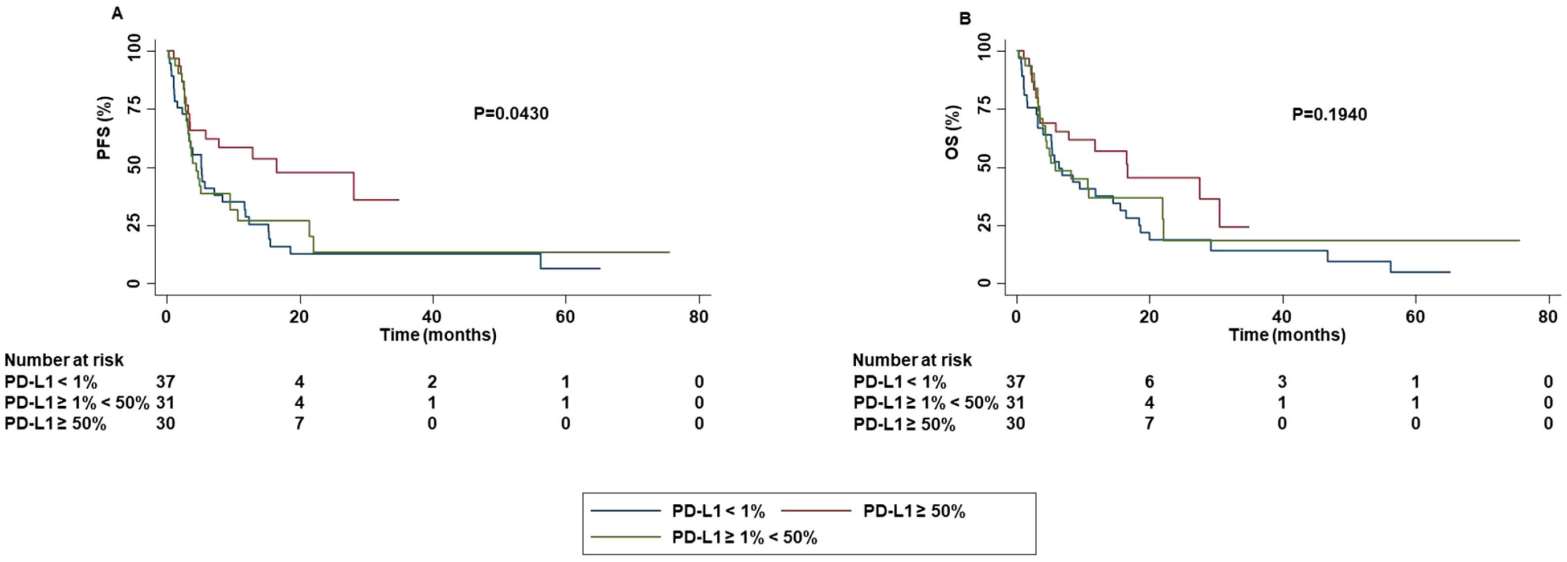
Association between PD-L1 TPS and clinical outcomes in advanced non oncogene NSCLC patients treated with ICI-based immunotherapy. PFS **(A)** and OS **(B)** of advanced NSCLC patients treated with ICI-based immunotherapy were stratified based PD-L1 TPS. PFS and OS were compared using the Kaplan-Meier method. Differences in patients’ survival were analyzed using a log-rang test. P <0.05 was considered statistically significant.

Correlation between PD-L1 TPS and survival outcomes showed that the risk of progression was significantly decreased for the patients with PD-L1 TPS ≥ 50% as compared to those with PD-L1 TPS < 50% (P=0.0430) (**Figure 9**).

**Figure 10:**
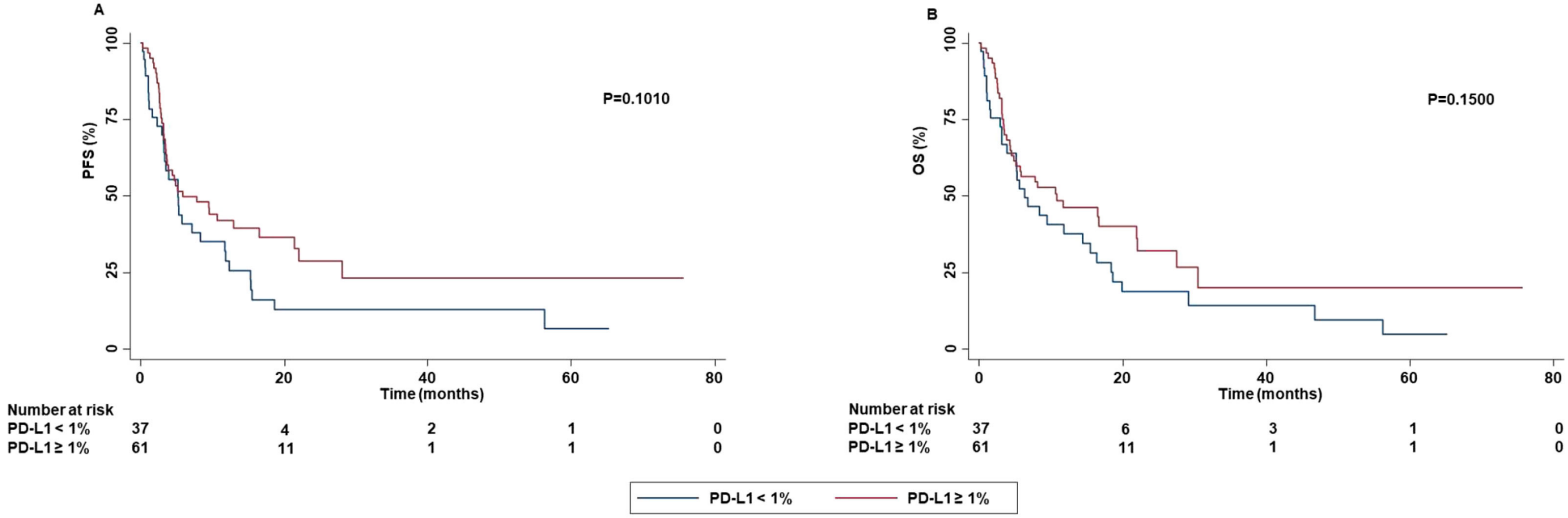
Association between PD-L1 TPS and clinical outcomes in advanced non oncogene NSCLC patients treated with ICI-based immunotherapy. PFS **(A)** and OS **(B)** of advanced NSCLC patients treated with ICI-based immunotherapy were stratified based on the absence (PD-L1 TPS < 1%) or the presence (PD-L1 TPS ≥ 1%) of PD-L1 TPS. PFS and OS were compared using the Kaplan-Meier method. Differences in patients’ survival were analyzed using a log-rang test. P <0.05 was considered statistically significant.

No significant difference in OS was detected based on PD-L1 TPS. In addition, a significantly higher ORR (P=0.0210) and DCR (P=0.0030) was obtained in patients with PD-L1 TPS > 1% or < 50% as compared to those with TPS ≥ 50%. Both patients with PD-L1 TPS < 1% or ≥ 50% achieved a lower rate of complete or partial response than those with TPS ≥ 1% and < 50%. Stratification of patients based on their negative (PD-L1 TPS < 1%) or positive (PD-L1 TPS ≥ 1%) value showed that patients with PD-L1 TPS ≥ 1% achieved a significant higher percentage of survival at 60 months as compared to those with PD-L1 TPS < 1% in both PFS and OS. Differences in median PFS and OS were not significant (PFS: P=0.1010; OS:P=0.1500) (**Figure 10**).

We also explored whether different treatment regimens influenced the survival outcomes. Patients treated with ICIs as first-line achieved numerically longer PFS and OS as compared to those treated with ICIs as second or subsequent lines. However, these differences are not statistically significant (PFS: P=0.1500 and OS: P=0.2300). In contrast, the differences in terms of survival outcomes were statistically significant when we considered the specific lines of treatment (first, second, third…) (PFS: P=0.039 and OS: P=0.0330). Lastly, patients treated with the combination of chemotherapy and ICIs achieved a numerically longer PFS and OS as compared to those treated with ICIs as monotherapy. However, these differences are not statistically significant (PFS: P=0.2800 and OS: P=0.5200).

**Figure 11:**
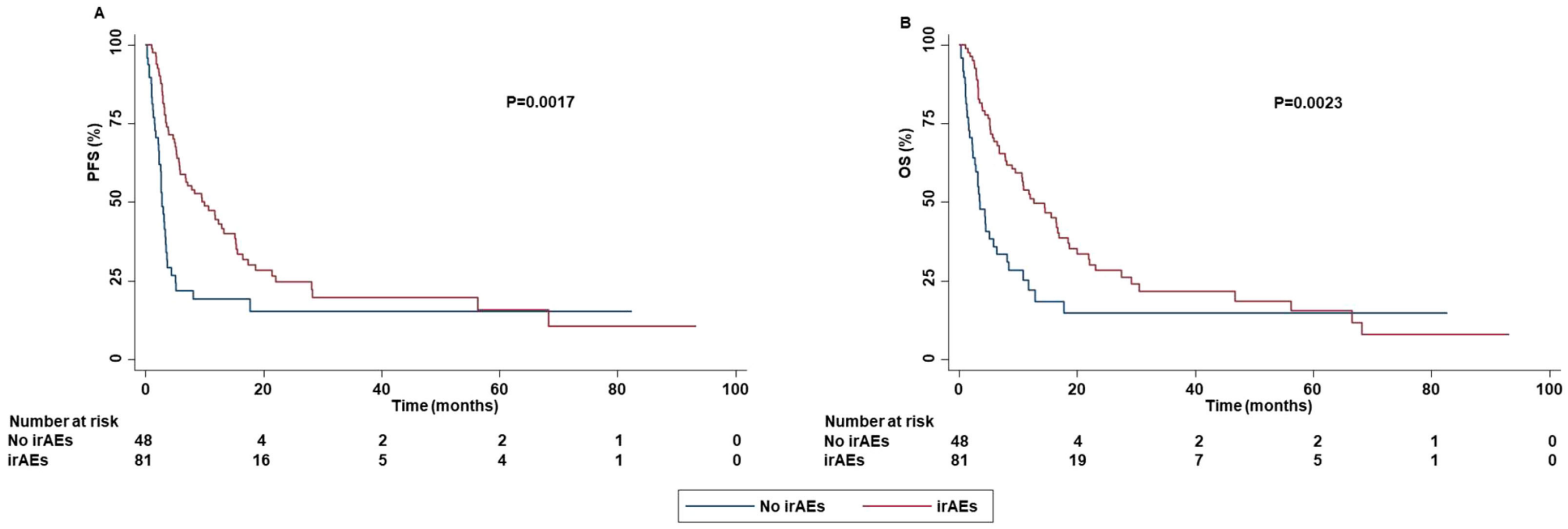
Association between the occurrence of irAEs and clinical outcomes in advanced non oncogene NSCLC patients treated with ICI-based immunotherapy. PFS **(A)** and OS **(B)** of advanced NSCLC patients treated with ICI-based immunotherapy were stratified based on the presence or absence of irAEs. PFS and OS were compared using the Kaplan-Meier method. Differences in patients’ survival were analyzed using a log-rang test. P <0.05 was considered statistically significant.

Concomitant administration of antiplatelet drugs, statin or low dose of aspirin was significantly correlated with the occurrence of irAEs. Specifically, patients who assumed antiplatelet drugs (P=0.0489) or statin (P=0.0400) had an increased risk to develop grade 3-4 irAEs as well as those who assumed low dose of aspirin had an increased risk to develop all grade irAEs (P=0.0016). In addition, the line of treatment was also associated with the occurrence of irAEs. Indeed, patients treated with ICI as first-line reported a lower rate of all grade (P=0.0348) and grade 1-2 (P=0.0329) irAEs than those treated with ICIs as second or subsequent-line of treatment. The occurrence of irAEs was significantly associated with survival outcomes. Specifically, the occurrence of all grade irAEs was significantly correlated with increased PFS (P=0.0017) and OS (P=0.0023) (**Figure 11**).

**Figure 12:**
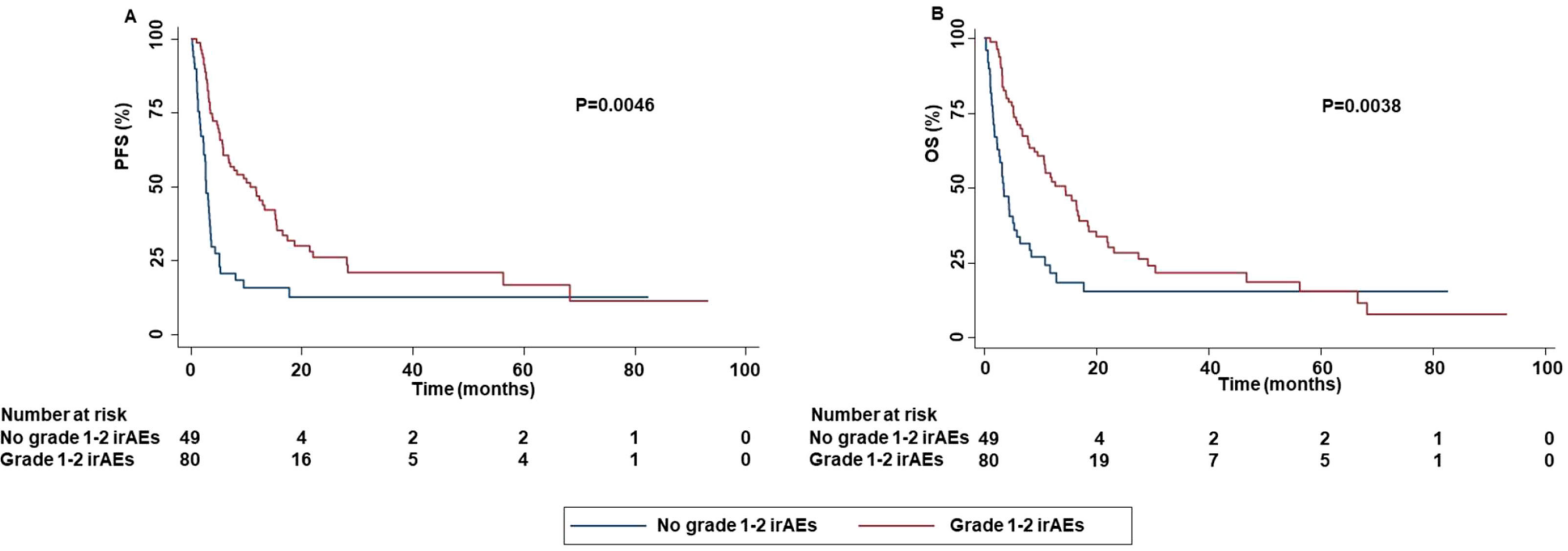
Association between grade 1-2 irAEs and clinical outcomes in advanced non oncogene NSCLC patients treated with ICI-based immunotherapy. PFS **(A)** and OS **(B)** of advanced NSCLC patients treated with ICI-based immunotherapy were stratified based on the occurrence of irAEs. PFS and OS were compared using the Kaplan-Meier method. Differences in patients’ survival were analyzed using a log-rang test. P <0.05 was considered statistically significant.

In addition, the occurrence of grade 1-2 irAEs was strongly correlated with an increased PFS (P=0.0046) and OS (P=0.0038) (**Figure 12**).

**Figure 13:**
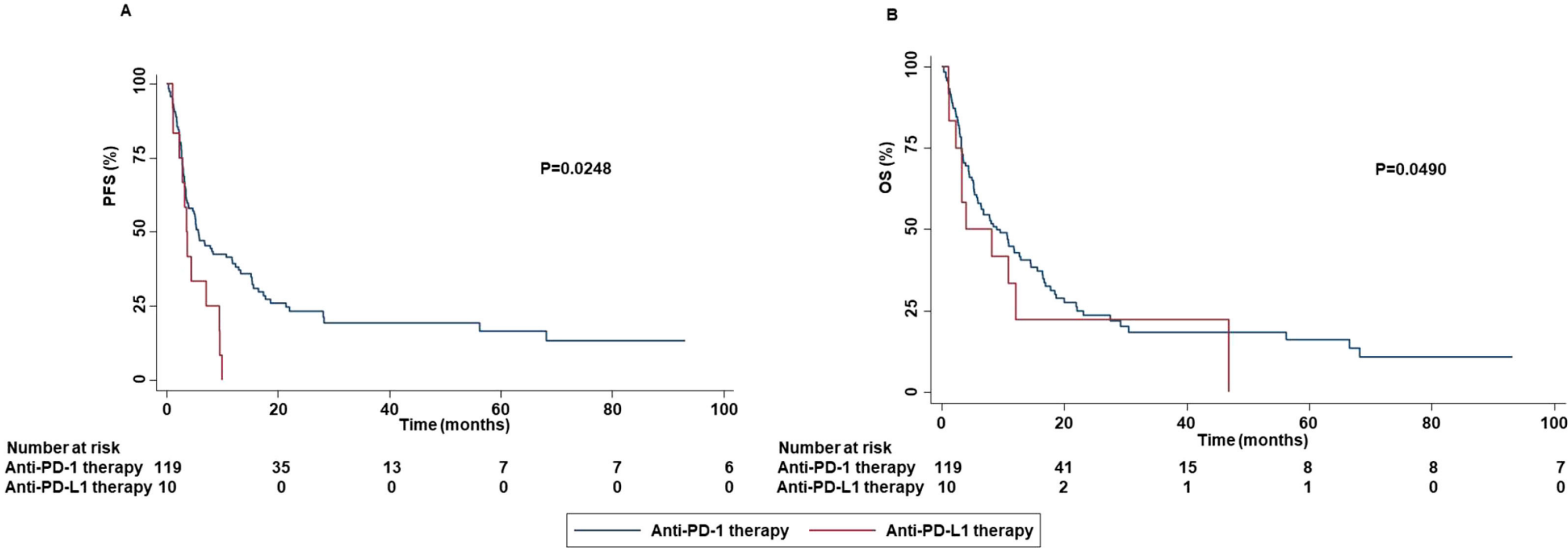
Association between type of ICI-based immunotherapy and clinical outcomes in advanced non oncogene NSCLC patients treated with ICI-based immunotherapy. PFS **(A)** and OS **(B)** of advanced NSCLC patients treated with ICI-based immunotherapy were stratified based on type of ICIs. Specifically, anti-PD-1-based immunotherapy versus anti-PD-L1-based immunotherapy. PFS and OS were compared using the Kaplan-Meier method. Differences in patients’ survival were analyzed using a log-rang test. P <0.05 was considered statistically significant.

These results were corroborated by the significant association between the occurrence of all grade and grade 1-2 irAEs with DCR (P=0.0021 and P=0.0017). No significant association between grade 3-4 irAEs and survival (PFS (P=0.6510) or OS (0.4971) (**Supplementary Figure S5**) or response outcomes ((ORR (P=0.8510) and DCR (0.2230)) was found. Analysis of potential correlation between type of ICI-based immunotherapy and survival outcomes showed that patients treated with anti-PD-1-based therapy achieved an increased PFS (P=0.0248) and OS (P=0.0490) than those treated with anti-PD-L1-based therapy (**Figure 13**).

**Figure 14:**
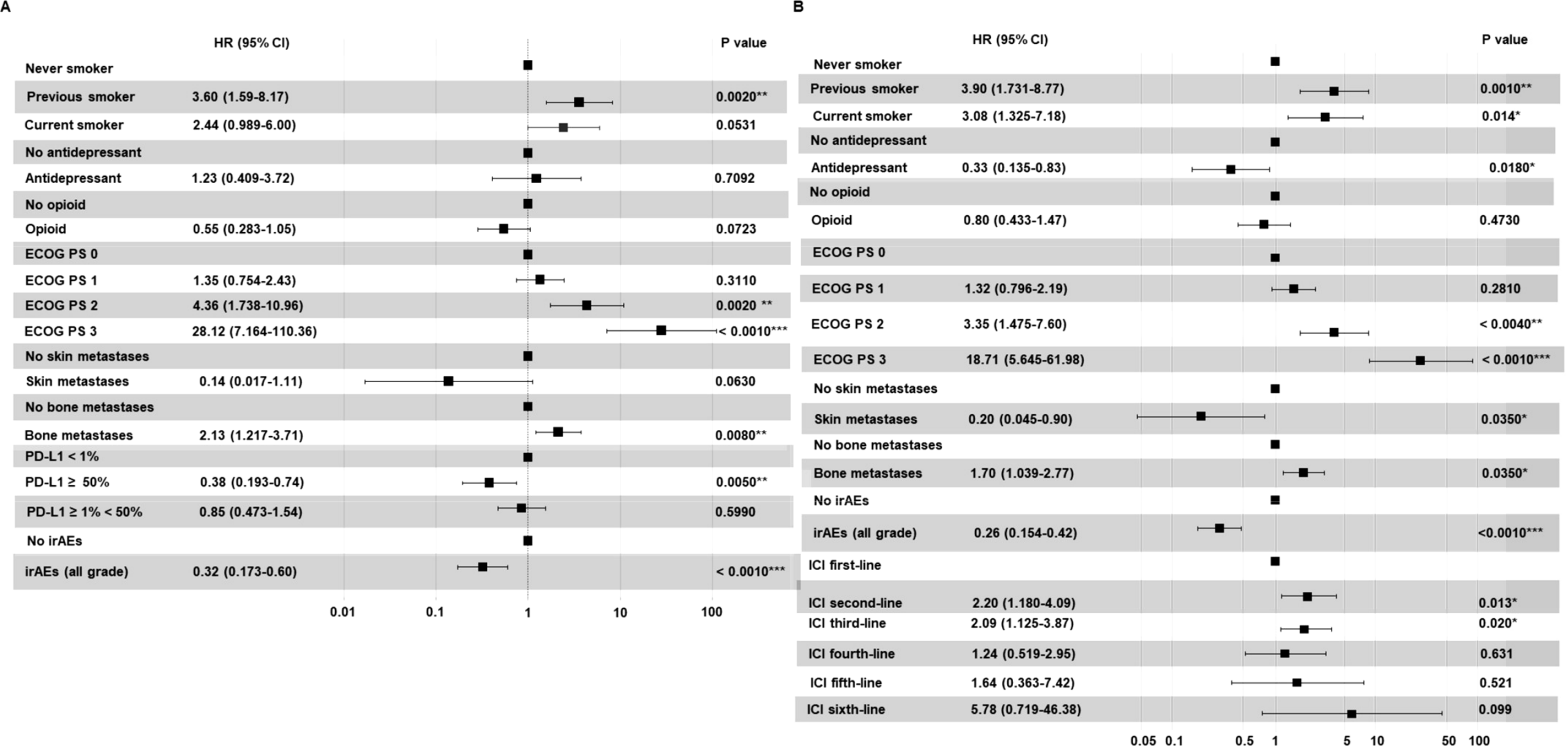
Multivariate analysis testing the correlation between clinical-pathological characteristics and PFS **(A)** or OS **(B)** in advanced non oncogene NSCLC patients treated with ICI-based immunotherapy. Multivariate survival analyses were performed using the Cox proportional hazards model. Symbols *,**,*** indicate P value < 0.05, 0.005, 0.001.

Nevertheless, no significant difference in terms of DCR (P=0.1530) or ORR (P=0.2100) based on the type of ICI-based immunotherapy (anti-PD-1-based therapy vs anti-PD-L1-based therapy) was found. Validation of the results obtained in univariate analysis by a multivariate analysis demonstrated that smoking status (P=0.0020), ECOG PS (P=0.0020), presence of bone metastases (P=0.0080), PD-L1 TPS (P=0.0050) and occurrence of all grade irAEs (P=0.0010)) significantly correlated with PFS (**Figure 14A**) while smoking status (P=0.0010), concomitant assumption of antidepressant (P=0.0180), ECOG PS (P=0.0010), presence of skin (P=0.0350) or bone (P=0.0350) metastases, the occurrence of all grade irAEs (P=0.0010) and the specific lines of treatment (P=0.020) were significantly correlated with OS (**Figure 14B**). No significant difference when we included the specific lines of treatment in the multivariate analyses for PFS was found (data not shown).

## Discussion

In the past few years, the efficacy of ICI-based immunotherapy for the treatment of advanced non oncogene NSCLC was demonstrated in many clinical trials (3–11). This evidence was validated in various real-world studies (24–27). In the present work, we reported a real-world experience of 129 patients with advanced non oncogene NSCLC treated with ICI-based immunotherapy. Median PFS and OS were 5.27 months and 8.43 months, respectively. These results are in line with those from other real-world experiences which included patients treated with ICI as second or subsequent-line of treatment (24, 26). In contrast, higher median PFS and OS were reported in other studies which only included patients treated with ICI as first-line of treatment (25, 28). Here, the study population included 100 male patients (77.52%) with a median age of 68 years. These results confirm a high prevalence of advanced non oncogene NSCLC in male and older patients (1). The latter are frequently affected by multiple comorbidities requiring appropriate concomitant medications. As a result, our study population allowed us to investigate whether comorbidities and concomitant medications may impact on the survival outcomes. At least one comorbidity was reported in 94 of the patients (75.78%). The high prevalence of comorbidities is consistent with that of a real-world population of NSCLC patients with a median age of 68 years. For instance, hypertension and COPD were reported in 53.49% and 17.05% of treated patients, respectively. Various studies have already investigated the potential predictive role of comorbidities in this subgroup of patients without unique conclusions (29–32). In our study no significant association between comorbidities and survival outcomes emerged in our study (**Supplementary Table S1**).

Survival analysis of subpopulation of patients treated with ICI as first-line yielded a median PFS and OS of 5.87 months and 11.80 months, respectively. Noteworthy, while median PFS is in line with data provided in clinical trials and other real-world studies, median OS resulted significantly decreased (25, 28, 33–36). This discrepancy might be explained by the i) the high percentage of comorbidities in our study population; ii) presence of poor prognosis-related clinical characteristics including ECOG PS 2-3 and bone/brain metastases.

On the other hand, among patients treated with ICI as second or subsequent lines of treatment, the median PFS and OS were 3.90 months and 8.12 months, respectively. These results are in line with those reported in the main clinical trials as well as other real-world experiences (4, 5, 37–39).

Survival analyses of the two subgroups of patients treated with the combination of chemotherapy and ICI or ICI as monotherapy were also performed. In the former, at a median follow-up of 23.40 months, median PFS and OS were 8.37 months and 11.80 months, respectively. According with results from the overall study population, median PFS was in line with data of clinical trials and other real-world experiences whereas median OS was significantly decreased (6, 11, 33, 40–42). Conversely, according with our data, in the study of *Verschueren* et al. a median OS of about 10 months was reported (43). In the latter subgroup, at a median follow-up of 69.80 months, the median PFS and OS were 4.43 months and 8.17 months, respectively. These results are globally lower than those reported in the literature (4, 5, 21, 25, 28, 31, 33, 34, 37, 38). However, a large portion of patients we have analyzed (about 70%) received ICI as monotherapy in second or subsequent-line of treatment explaining this difference. Overall, further studies are needed to predict more accurately the survival benefit of ICI-based immunotherapy in the real-world populations of advanced non oncogene NSCLC patients.

Toxicity analysis was also performed in our study. Grade 1-2 and grade 3-4 irAEs were reported in 62.02% and 9.38% of treated patients, respectively being in line with data of the literature (3–11). Furthermore, in order to validate the quality of our study population, we demonstrated that patients with complete or partial response, achieved better survival outcomes and received an increased number of ICIs than those with stable or progressive disease. Noteworthy, we reported that age of the patients may influenced the choice of the type of treatment by the clinical oncologists. Specifically, older patients were treated more frequently with ICI as monotherapy than the combination of chemotherapy and ICI (P=0.0480). This result may be explained by the worse toxicity-profile of the combination than monotherapy (6, 11, 21, 44). However, the sample size of our study population was not enough to demonstrate this association (effect size: 0.0473; power: 0.0832). Larger studies are needed to validate this hypothesis. On the same line, our older patients were treated more frequently with anti-PD-L1 therapy as compared to anti-PD-1 therapy. This difference reflected the general idea of the better toxicity-profile of anti-PD-L1 therapy than anti-PD-1 therapy. This issue was investigated by many studies with contrasting results (45–48). Some of them have shown an higher rate of irAEs for anti-PD-1 therapy while no difference was found in others (45–48). In our work, no difference in terms of toxicity was found between patients treated with anti-PD-1 therapy than those treated with anti-PD-L1 therapy.

Although ICI-based immunotherapy represents the cornerstone of the treatment of patients with advanced non-oncogene NSCLC, almost half of treated patients did not obtain any clinical benefit from this novel immunotherapeutic approach (3–11). Our study confirmed this crucial issue. Indeed, about 40% of treated patients achieved a radiologic progression of disease as best response. Then, predictive biomarkers of tumor response are urgently needed to improve the therapeutic algorithms of these patients. We found that specific clinical-pathological characteristics including ECOG PS, smoking status, concomitant administration of antidepressants or opioids, bone metastases, skin metastases, PD-L1 TPS, and the occurrence of irAEs influenced the survival outcomes.

According to our findings, the predictive role of specific clinical-pathological characteristics was already investigated with various results (20, 26). Robust data have already demonstrated that patients with ECOG PS ≥ 2 presented worse survival outcomes than those with ECOG PS < 2 (20, 49, 50). The high tumor load and/or the presence of multiple concomitant comorbidities may explain this difference. In our study no correlation between tumor load or concomitant comorbidities and ECOG PS was demonstrated. However, the presence of comorbidities was not significantly associated with survival outcomes.

The predictive role of smoking status was also investigated with contrasting results. For instance, *Wang* et al. demonstrated that increased smoking exposure was significantly correlated with improved clinical benefit, regardless PD-L1 status (51). Conversely, *Chen* et al, in the conclusions of their meta-analysis suggested that smoking status should not be recognized as predictive of ICI-based immunotherapy in patients with advanced NSCLC (52). Beyond clinical results, smoking is well-known to be associated with increased TMB (53) which is in turn correlated with higher likelihood to achieve a clinical benefit from ICIs (54). As a result, it seems reasonable to think that smoking status may influence tumor response. In our study, we did not investigate the role of TMB and its potential correlation with smoking status and/or tumor response. However, the smoking status significantly correlated with PFS and OS. Indeed, never smokers displayed increased PFS and OS than current and previous smokers. In addition, patients who discontinued smoking, achieved decreased PFS than current smokers (this effect is not influenced by age (p=0.4460) and comorbidities of the patients (**Supplementary Table S2**). These results were also confirmed by multivariate analyses. However, further studies are needed to clarify the predictive role of smoking status in advanced non oncogene addicted NSCLC patients treated with ICIs.

The potential predictive role of concomitant medications was also investigated. *Sieber* et al. have shown no significant correlation between concomitant medications such as metformin, antihypertensive and low dose of aspirin and survival outcomes (55). In contrast, concomitant assumption of antibiotics, proton pump inhibitors (PPIs), anticoagulants and opioids was associated with worse survival outcomes (56). In our study, at the univariate analyses, concomitant administration of antidepressants was correlated with increased PFS and OS whereas opioids with decreased PFS and OS. Other studies have already reported the negative predictive role of concomitant assumption of opioids (56–58). However, whether this effect is directly mediated by the opioids or reflects the presence of other negative clinical factors such as the presence of bone metastases or poor ECOG PS should be clarified. Some evidence suggested the negative predictive role of concomitant administration of opioids is caused by the modifications of the gut microbiome induced by their chronic assumption (59, 60). In our population, the concomitant assumption of opioids was significantly associated with the presence of bone metastases (P=0.023) and/or poor ECOG PS (P=0.000). In addition, at the multivariate analyses, no statistically significant association between concomitant administration of opioids and survival outcomes were found. As a result, our findings suggested that the predictive significance of opioids should be further investigated.

On the other hand, few studies have investigated the potential impact of the concomitant administration of antidepressants in advanced non oncogene NSCLC patients treated with ICIs. Preclinical studies have suggested that monoamine oxidase A (MAO-A) inhibitors and SSRIs may influence the tumor microenvironment exerting a synergistic cytotoxic effect with anti-PD-1 therapy on cancer cells (61–63). However, in our study, while the multivariate analysis confirmed the statistically significant correlation between the concomitant administration of antidepressants and OS, no correlation with PFS was found. This discrepancy may be the results of the different subsequent therapies and their potential synergistic effects with the concomitant administration of antidepressants. Further studies should clarify the predictive role of antidepressants in these patients as well as the underlying biological mechanisms. The latter should open the door to develop novel synergistic therapeutic strategies.

Beyond concomitant medications, specific sites of metastasis may also influence the efficacy of ICI-based immunotherapy in this patient population (64–66). In multivariate analyses, the presence of bone metastases was significantly correlated with worse PFS and OS. In line with other studies (67–69), our results confirmed the independent negative predictive role of the bone metastases in patients with advanced non oncogene NSCLC. The “cold” tumor microenvironment of NSCLC patients with bone metastases may explain their negative predictive role (67). A deeper knowledge of the characteristics of tumor microenvironment is crucial to build potential strategies to improve the survival of these patients.

Conversely, in the multivariate analysis, the presence of skin metastases was significantly correlated with better OS. The presence of skin metastases was also numerically correlated with better PFS without reaching the statistically significance at multivariate analysis (P=0.063). To the best of our knowledge, no other study has demonstrated this type of association. The latter was independent by the presence/absence of other sites of metastases (**Supplementary Table S3**). In addition, all six patients with skin metastases presented at least another site of metastasis. However, in our study population, the presence of skin metastases was reported in only six patients. As a result, larger studies are needed to confirm this type of association.

As mentioned above, among several predictive biomarkers, PD-L1 TPS is the most widely investigated (13, 17–19). To date, many issues about the utilize of PD-L1 TPS as predictive biomarker remain unsolved and the evidence available has provided very heterogenous results (13, 17–19). In our study, patients with PD-L1 TPS < 50% had a shorter PFS than those with PD-L1 TPS ≥ 50% (P=0.0430). This result was confirmed by multivariate analysis, although no significant correlation between PD-L1 TPS and OS was found. This discrepancy has clinical relevance confirming the scarce predictive value of PD-L1 TPS.

In line with other studies (70–74), the occurrence of all grade irAEs was significantly correlated with increased PFS and OS. We have to note that the presence of immortal-time bias in our study population might limit the validity of this result (75). On the other hand, the statistical significance at the multivariate analysis as well as the correlations between the occurrence of irAEs and both ORR and DCR, corroborated this finding. In contrast, other studies have reported no significant association between the occurrence of irAEs and survival outcomes (16, 76). As a result, this correlation, and the underlying biological mechanisms should be further investigated. In addition, it remains also unknown how clinical oncologists may utilize this correlation to improve the therapeutic algorithm of these patients.

Lastly, we explored potential correlations between clinical-pathological characteristics and irAEs. Noteworthy, in patients who assumed antiplatelet drugs or statin we reported a higher rate of grade 3-4 irAEs. Moreover, in those assuming low dose of aspirin a higher rate of all grade irAEs was reported. This correlation has clinical relevance since a high percentage of patients with advanced NSCLC assumed these drugs. Various studies have already investigated whether concomitant medications influenced the occurrence of irAEs with contrasting results. For instance, *Yang* et al, demonstrated the correlation between the use of aspirin and the occurrence of irAEs in a pan-cancer study population (77). In contrast, *Kostine* et al, showed that use of aspirin, antibiotics, glucocorticoids, PPIs, opioid, non-steroidal anti-inflammatory drugs (NSAIDs), and psychotropic drugs was associated with decreased occurrence of irAEs (78). Further studies are needed to clarify this difference as well as the potential implications in the clinical management of this patient population.

In our study, patients treated with ICIs as first-line reported a lower rate of all grade and grade 1-2 irAEs as compared to those treated with ICIs as second or subsequent lines. This correlation suggests that previous treatments may influence the predisposition to irAEs. To the best of our knowledge, no study demonstrated the biological mechanisms underlying this type of association. However, we have to highlight that all patients with advanced non oncogene NSCLC received ICI in the first-line setting as monotherapy or in combination with chemotherapy, so far. As a result, although of biological interest, this finding has no high clinical relevance.

## Conclusions

In this study we reported our real-world experience of ICIs for the treatment of patients with advanced non oncogene NSCLC. Decreased clinical benefit in terms of overall survival as compared to that of patients included in the clinical trials was reported in our study population. However, in the specific subgroup of patients treated with ICI as monotherapy in second or subsequent-line setting, our results are in line with clinical trials as well as other real-world experiences. Noteworthy, some correlations between clinical-pathological characteristics and survival outcomes emerged. However, we have to highlight that these correlations emerged from a retrospective analysis and should be read for generating hypotheses. The potential integration of clinical-pathological characteristics in more accurate predictive algorithms as well as the underlying biological mechanisms should be further validated in *ad hoc* prospective studies.

## Data Availability

The raw data supporting the conclusions of this article will be made available by the authors, without undue reservation.
