# Supplementary material for: Checkpoint based immunotherapy in non-small cell lung cancer: a real-world retrospective study
Source: Front Immunol. 2024 Nov 27;15:1419544. doi: 10.3389/fimmu.2024.1419544 (PMC11631946; doi:10.3389/fimmu.2024.1419544)
Supplement: Supplementary file 1 [file DataSheet1.docx]

Supplementary Material


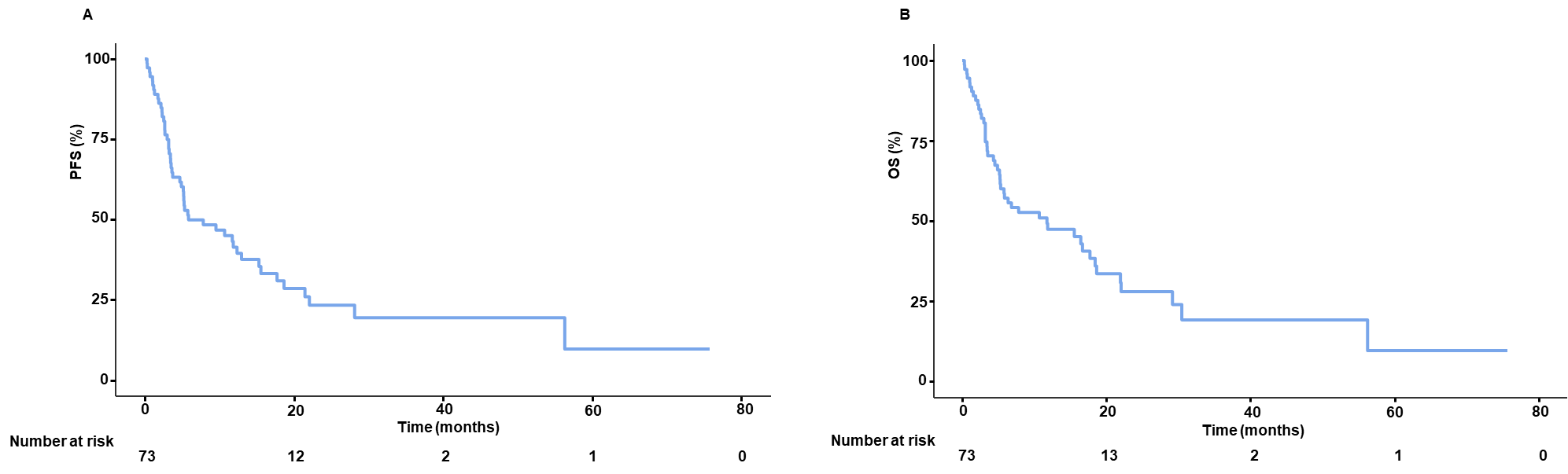


**Supplementary Figure 1 PFS and OS of advanced non oncogene NSCLC patients treated with ICI-based immunotherapy as first-line.** At a median follow-up of 21.30 months, median PFS and OS were 5.87 months (range, 0.30-75.73 months) months (**A**) and 11.80 months (range, 0.30-75.73 months) (**B**), respectively. PFS and OS analysis was performed using the Kaplan-Meier method.


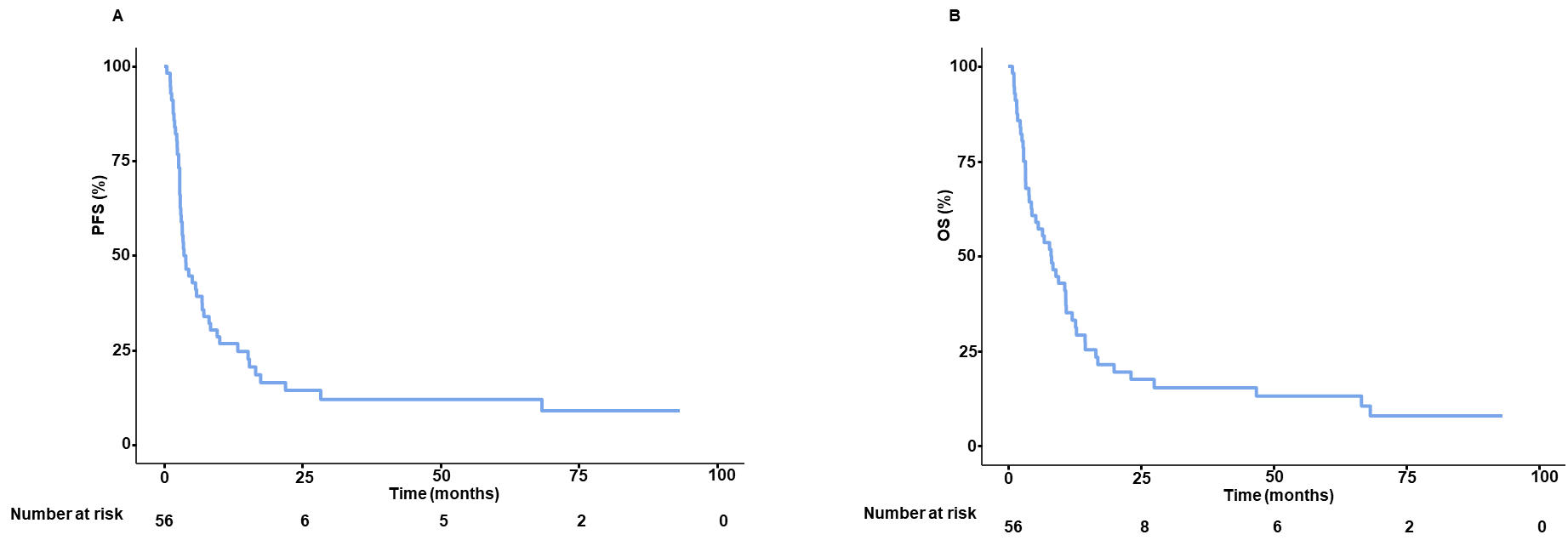


**Supplementary Figure 2 PFS and OS of advanced non oncogene NSCLC patients treated with ICI-based immunotherapy as second or subsequent-line.** At a median follow-up of 69.80 months, median PFS and OS were 3.90 months (range, 0.47-93.23 months) (**A**) and 8.12 months (range, 0.80-93.23 months) (**B**), respectively. PFS and OS analysis was performed using the Kaplan-Meier method.


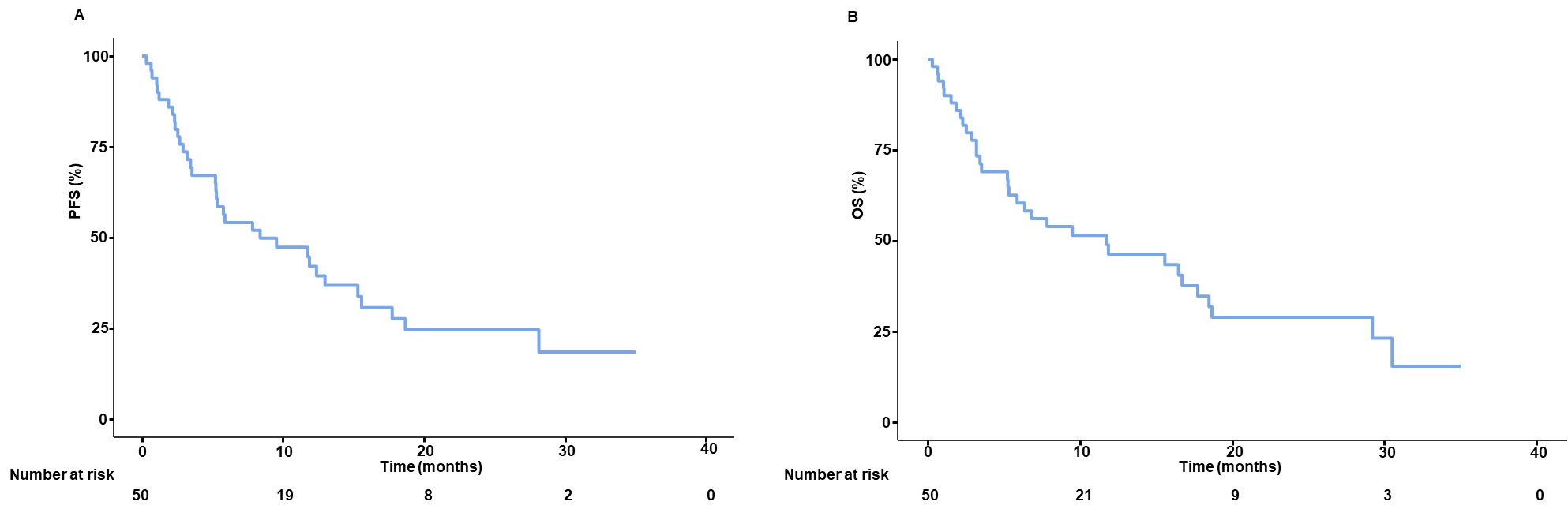


**Supplementary Figure 3 PFS and OS of advanced non oncogene NSCLC patients treated with the combination of chemotherapy and ICI.** At a median follow-up of 23.40 months, median PFS and OS were 8.37 months (range, 0.30-35.00 months) (**A**) and 11.80 months (range, 0.30-35.00 months) (**B**), respectively. PFS and OS analysis was performed using the Kaplan-Meier method.


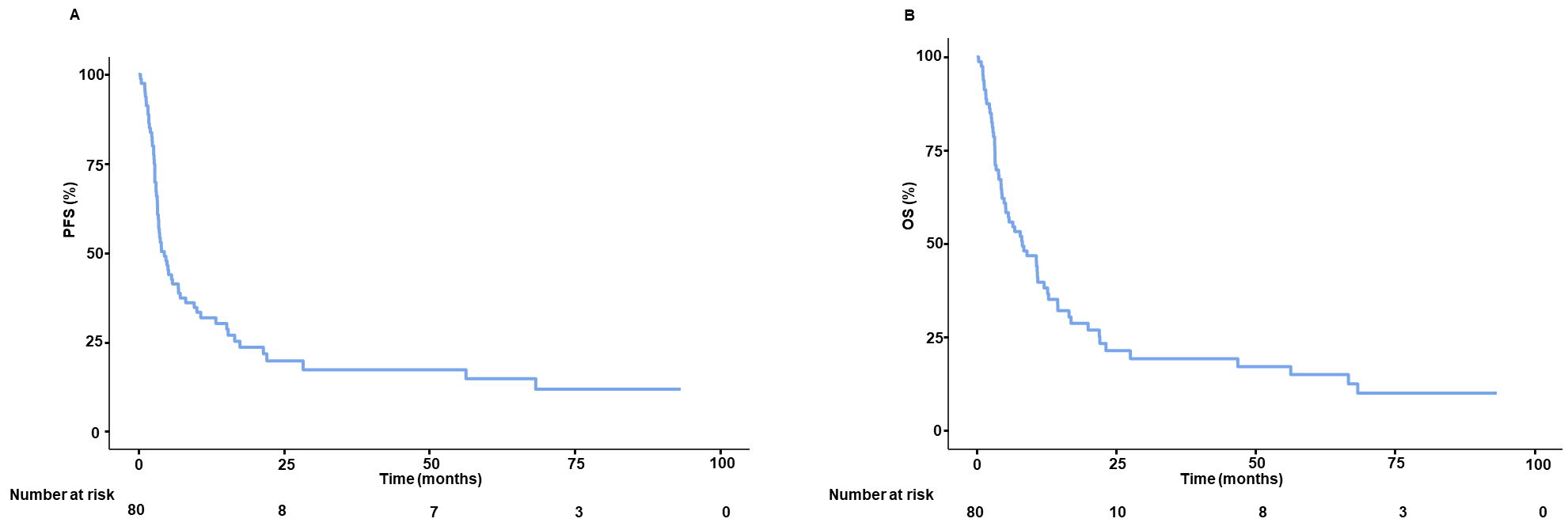


**Supplementary Figure 4 PFS and OS of advanced non oncogene NSCLC patients treated with ICI as monotherapy.** At a median follow-up of 69.80 months, median PFS and OS were 4.43 months (range, 0.33-93.23 months) (**A**) and 8.17 months (range, 0.33-93.23 months) (**B**), respectively. PFS and OS analysis was performed using the Kaplan-Meier method.


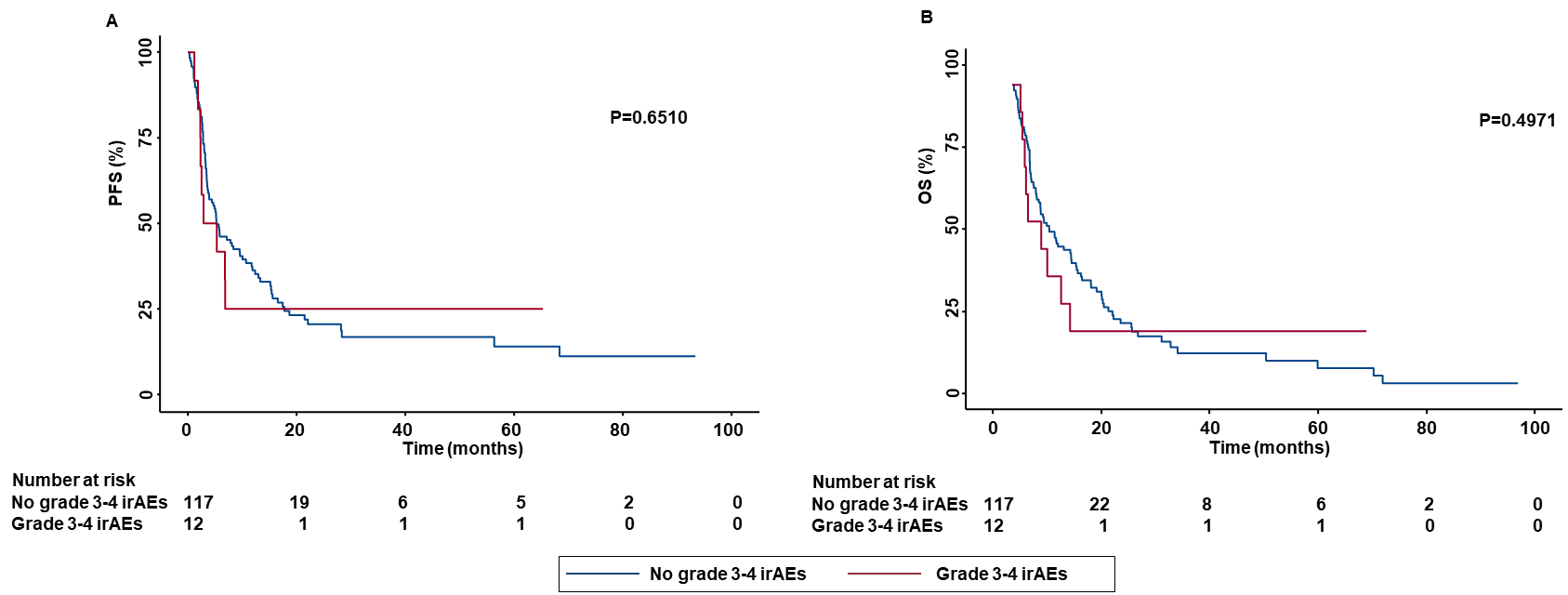


**Supplementary Figure 5 Association between grade 3-4 irAEs and clinical outcomes in advanced non oncogene NSCLC patients treated with ICI-based immunotherapy.** PFS **(A)** and OS **(B)** of advanced NSCLC patients treated with ICI-based immunotherapy were stratified based on the occurrence of irAEs. PFS and OS were compared using the Kaplan-Meier method. Differences in patients’ survival were analyzed using a log-rang test. P <0.05 was considered statistically significant.

**Supplementary Table 1 Univariate analysis testing the potential association between comorbidities and survival outcomes**

| **Comorbidity** | **PFS**  **HR (95% CI) P value** | | | **OS**  **HR (95% CI) P value** | |
| --- | --- | --- | --- | --- | --- |
| **Hypertension** | 1.30 (0.86-1.96) | | 0.2090 | 1.10 (0.73-1.66) | 0.6500 |
| **Dyslipidemia** | 0.99 (0.64-1.54) | 0.9630 | | 1.01 (0.65-1.59) | 0.9570 |
| **Diabetes** | 0.74 (0.45-1.23) | 0.2480 | | 0.75 (0.45-1.26) | 0.2800 |
| **COPD** | 1.37 (0.83-2.27) | 0.2190 | | 1.29 (0.77-2.16) | 0.3370 |
| **HF** | 0.62 (0.27-1.42) | 0.2600 | | 0.68 (0.30-1.57) | 0.3690 |
| **Depressive disorder** | 0.44 (0.11-1.78) | 0.2490 | | 0.45 (0.11-1.85) | 0.2700 |

**Supplementary Table 2 Distribution of comorbidities in current smokers versus previous smokers.**

| **Comorbidity** | **Previous Smokers** | **Current Smokers** | **P value** |
| --- | --- | --- | --- |
| **Hypertension**  Yes  No | 45  30 | 18  19 | 0.2550 |
| **Dyslipidemia**  Yes  No | 26  49 | 9  28 | 0.2670 |
| **Diabetes**  Yes  No | 14  62 | 11  26 | 0.1740 |
| **COPD**  Yes  No | 15  61 | 7  30 | 0.9180 |
| **HF**  Yes  No | 7  69 | 3  34 | 0.8460 |
| **Depressive disorder**  Yes  No | 2  74 | 1  36 | 0.9820 |
| **CRF**  Yes  No | 0  74 | 0  37 | * |

*No CRF in both current and previous smokers was reported.

**Supplementary Table 3 Distribution of other sites of metastases in patients with or without skin metastases.**

| **Other site of metastases** | **Skin Metastases** | **No Skin Metastases** | **P value** |
| --- | --- | --- | --- |
| **Lymph node**  Yes  No | 6  0 | 106  16 | 0.3430 |
| **Lung**  Yes  No | 3  3 | 81  41 | 0.4090 |
| **Bone**  Yes  No | 2  4 | 36  86 | 0.8410 |
| **CNS**  Yes  No | 2  4 | 26  96 | 0.4870 |
| **Adrenal gland**  Yes  No | 1  5 | 22  100 | 0.9320 |
| **Liver**  Yes  No | 0  6 | 15  107 | 0.3610 |

**Supplementary Results 1: Effect size and power for P value = 0.05 correlations**

| **Type of correlation** | **Effect size** | **Power** |
| --- | --- | --- |
| Age-Type of therapy | 0.0473 | 0.0832 |
| Opioids-ORR | 0.4670 | 1.000 |
| Skin metastasis-ORR | 0.4650 | 0.999 |

**Supplementary Results 2: Post hoc pairwise analysis for correlating age to the type of therapy**

| **Type of therapy** | **W** | **P value** |
| --- | --- | --- |
| IT vs CT+IT | -2.77 | 0.1230 |
| IT vs CT+IT+IT | -2.41 | 0.2040 |
| CT+IT vs CT+IT+IT | -1.58 | 0.5030 |
| Anti-PD-1 vs anti-PD-L1 | -2.82 | 0.4610 |
